# Integration of Transcriptomics and Metabolomics for Understanding the Different Vegetative Growth in *Morchella Sextelata*

**DOI:** 10.3389/fgene.2021.829379

**Published:** 2022-02-04

**Authors:** Kejun Deng, Xiuhua Lan, Ying Chen, Ting Wang, Mengke Li, Yingyin Xu, Xuelian Cao, Guangbo Xie, Liyuan Xie

**Affiliations:** ^1^ School of Life Science and Technology, Center for Informational Biology, University of Electronic Science and Technology of China, Chengdu, China; ^2^ Institute of Agricultural Resources and Environment, Sichuan Academy of Agricultural Sciences, Chengdu, China

**Keywords:** regulation mechanism, pathway, transcriptomics, metabolomics, nutritional composition, cultivation

## Abstract

*Morchella sextelata* is an edible and medicinal fungus with high nutritional, medicinal, and economic value. Recently, *M*. *sextelata* has been produced through artificial cultivation in China, but its stable production remains problematic because the details of its growth and development process are limitedly understood. Herein, to investigate the dynamic process of *M*. *sextelata* development, we integrated the transcriptomics and metabolomics data of *M. sextelata* from three developmental stages: the young mushroom period (YMP), marketable mature period (MMP), and physiological maturity period (PMP). The results showed that the transcriptome changed dynamically at different stages and demonstrated the significant enrichment of pathways that regulate plant growth and development, such as N-glycan biosynthesis and carbon and purine metabolism. Similarly, small-molecule metabolites, such as D-fructose-1,6-biphosphate, which was upregulated during the YMP, dihydromyricetin, which was upregulated during the MMP, and L-citrulline, which was upregulated during the PMP, also showed phase-dependent characteristics. Then, combined analysis of the transcriptome data and metabolome traits revealed that the transcriptome may affect metabolic molecules during different growth stages of *M. sextelata via* specific enzymes, such as α-glucosidase and glucanase, which were included in two opposite transcriptome modules. In summary, this integration of transcriptomics and metabolomics data for understanding the vegetative growth of *M. sextelata* during different developmental stages implicated several key genes, metabolites, and pathways involved in the vegetative growth. We believe that these findings will provide comprehensive insights into the dynamic process of growth and development in *M. sextelata* and new clues for optimizing the methods for its cultivation application.

## Introduction

The genus *Morchella* comprises edible and medicinal macrofungi, also known as morels because they have a structure similar with the upper cover. The members of this genus are highly appreciated for their nutraceutical, pharmaceutical, and economic importance and good taste (Tietel and Masaphy, 2018) and have been used as a traditional herbal medicine for centuries (Mau et al., 2004). Like most edible fungi, *Morchella* spp. contain high levels of unsaturated fatty acids, and are rich in a variety of mineral elements, vitamins, fresh substances, aromatic components, and bioactive components (Liu et al., 2016). In addition, numerous studies regarding the medicinal efficacy of *Morchella* spp. have mainly focused on their immune-regulatory, anti-fatigue, anti-cancer, hepatoprotective, and blood pressure-lowering abilities (Du et al., 2015; Liu et al., 2016; He et al., 2017; Liu et al., 2018). Furthermore, there is a premium demand for *Morchella* spp. among suppliers and consumers (Stott and Mohammed, 2004); the annual sale of *Morchella* spp. likely ranges from $5 million to $10 million in western North America (Pilz et al., 2007).

Due to the limited natural yield of wild morels, many attempts have been made for their artificial cultivation over the years. Moreover, a few species of *Morchella* have been successfully domesticated in America and China in recent years (Masaphy, 2010), including *M. sextelata* (Liu et al., 2020). The genetic and biological characteristics of *M*. *sextelata*, such as the mechanisms underlying its fruit formation, metamorphosis of its sclerotia, and exogenous nutrition, remain poorly understood; furthermore, spore formation at different stages of its life cycle has been reported to restrict its healthy development (Liu et al., 2015). Further, there are only a few studies regarding the developmental process of *M. sextelata*. Therefore, as *M. sextelata* is a rare and difficult to cultivate fungus, it is necessary to explore and analyze the molecular dynamics of its growth and development more comprehensively.

In this study, to elucidate the dynamic molecular panorama of the transcription and metabolism of *M. sextelata* during its growth, we integrated the transcriptomics and metabolomics data from three developmental stages (the young mushroom period (YMP), marketable mature period (MMP), and physiological maturity period (PMP)); these data are based on morphological and developmental changes in this fungus. We identified the key genes, metabolites, and pathways associated with its vegetative growth and then investigated the associated signal cascades. Overall, this study may help us understand the metabolic mechanisms and nutritional composition of *M. sextelata* and optimize the conditions for its cultivation.

## Materials and Methods

### Fungi and Growth Conditions

*M. sextelata* was cultivated using exogenous nutrient bags, which contained 100% straw or 85–90% straw and 15–10% husk, mixed with 1% lime and 1% gypsum. During the cultivation process, the soil was kept moist, the relative humidity in the shed was controlled between 85 and 90%, and the temperature was in the range of 8–20°C, ensuring that ventilation and “half shade and half sunlight” were maintained.

The fruiting body of this fungus shows a smooth cover, an unfolded ridge, a light-gray or gray-brown color, and a slightly expanded cylinder during the YMP. When the fruiting body caps grow to 3–6 cm in size, they can be sold as fresh mushrooms in the market; this stage was named the MMP. During the PMP, the color of the fruiting body’s cap is reddish-brown to dark reddish-brown, the cap layer is pale-pink, and the stipe is smooth, white, and trapezoid.

### RNA Extraction and Sequencing

Nine samples were collected for transcriptome sequencing (three samples in each stage). Total RNA was extracted using the TRIzol Reagent (Invitrogen) and purified using the Plant RNA Purification Reagent (Invitrogen), as described by the manufacturer. A total of 1 µg of RNA per sample was used as input material for the RNA sample preparations. Sequencing libraries were generated using the NEBNext^®^ UltraTM RNA Library Prep Kit for Illumina^®^ (NEB, United States), following the manufacturer’s recommendations; index codes were added to attribute sequences to each sample.

The clustering of the index-coded samples was performed on a cBot Cluster Generation System using TruSeq PE Cluster Kit v3-cBot-HS (Illumia), according to the manufacturer’s instructions. After cluster generation, the prepared libraries were sequenced on an Illumina Novaseq platform and 150-bp paired-end reads were generated.

### RNA-Sequence Analysis

Index of the reference genome was built and paired-end clean reads were aligned to the reference genome using Hisat2 v2.0.5 (Kim et al., 2015). The mapped reads of each sample were assembled using StringTie v1.3.3b (Pertea et al., 2015) *via* a reference-based approach. FeatureCounts v1.5.0-p3 (Liao et al., 2014) was used to count the numbers of reads mapped to each gene. Next, the expected number of FPKM (Fragments Per Kilo base of transcript sequence per Millions of base pairs sequenced) of each gene was calculated on the basis of the length of the gene and the number of reads mapped to this gene.

### Metabolite Extraction

A total of eighteen samples were collected for metabolomics, with six samples in each stage. Tissues (100 mg) were individually ground with liquid nitrogen and the homogenate was resuspended in 500 μl of prechilled 80% methanol and 0.1% formic acid, followed by thorough vortexing. The samples were then incubated on ice for 5 min and then centrifuged at 15,000 rpm (at 4°C) for 10 min. A portion of the supernatant was diluted with LC-MS-grade water to yield a final methanol concentration of 53%. The samples were subsequently transferred to a fresh Eppendorf tube and were then centrifuged at 15,000 *g* (at 4°C) for 20 min. Finally, the supernatant was injected into the LC-MS/MS system. Equal volumes of each experimental sample were taken and blended as quality control (QC) samples. The blank sample used was an aqueous solution of 53% methanol containing 0.1% formic acid (instead of the experimental sample); the pretreatment process for the blank sample was the same as that for the experimental sample. Note: The liquid samples (100 μl each) and prechilled methanol (400 μl) were mixed *via* thorough vortexing. The cell sample (50 μl each) and prechilled 80% methanol (200 μl) were mixed *via* thorough vortexing, followed by sonication for 6 min. This step was repeated once again, and then, the steps followed were the same as those mentioned above.

### HPLC-MS/MS Analysis

#### Positive-Ion Mode

The LC-MS/MS analyses were performed using an ExionLC™ AD system (SCIEX) coupled with a QTRAP^®^ 6,500 + mass spectrometer (SCIEX). The samples were injected into a BEH C8 Column (100 × 2.1 mm; 1.9 μm) using a 30 min linear gradient at a flow rate of 0.35 ml/min for the positive-polarity mode. The eluents used were eluent A (0.1% formic acid-water) and eluent B (0.1% formic acid-acetonitrile). The solvent gradient was set as follows: 5% B, 1 min; 5–100% B, 24.0 min; 100% B, 28.0 min; 100–5% B, 28.1 min; 5% B, 30 min. The QTRAP^®^ 6,500 + mass spectrometer was operated in the positive-polarity mode with a curtain gas setting of 35 psi, collision gas setting of medium, ion spray voltage of 5,500 V, temperature of 500°C, and ion source gas ratios of 1:55 and 2:55.

### Negative-Ion Mode

The samples were injected onto a HSS T3 Column (100 mm × 2.1 mm in size) using a 25 min linear gradient at a flow rate of 0.35 ml/min for the negative-polarity mode. The eluents used were eluent A (0.1% formic acid-water) and eluent B (0.1% formic acid-acetonitrile). The solvent gradient was set as follows: 2% B, 1 min; 2–100% B, 18.0 min; 100% B, 22.0 min; 100–5% B, 22.1 min; 5% B, 25 min. The QTRAP^®^ 6,500 + mass spectrometer was operated in the positive-polarity mode with a curtain gas setting of 35 psi, collision gas setting of medium, ion spray voltage of −4,500 V, temperature of 500°C, and ion source gas ratios of 1:55 and 2:55.

### Metabolite Identification and Quantification

The detection of the experimental samples using MRM (multiple reaction monitoring) was based on the novo gene in-house database. The Q3 quadrant was used for metabolite quantification. The following parameters were used for metabolite identification: Q1 quadrant, Q3 quadrant, RT (retention time), declustering potential (DP), and collision energy (CE). The data files generated by the HPLC-MS/MS analysis were processed using the SCIEX OS Version 1.4 to integrate and correct the peaks. The main parameters were set as follows: minimum peak height, 500; signal/noise ratio, 10; and Gaussian smooth width, 3. The area of each peak represents the relative content of the corresponding substance.

### Statistical Analysis

Differential expression analysis of two conditions was performed using the DESeq2 R package v1.16.1 (Love et al., 2014). The resulting *p*-values were adjusted using the Benjamini and Hochberg’s approach (adjusted *p*-value). We used the clusterProfiler (Yu et al., 2012) module of the R package to test the statistical enrichment of differentially expressed genes (DEGs) in the KEGG pathway analysis. Using EBSeq-HMM(Leng et al., 2015), we obtained the differentially expressed metabolites and their corresponding expression paths (i.e., “Up–Down”). Further, the relationship between expression abundance and time was calculated using the spearman algorithm in psych R package v2.1.3. The fuzzy clustering method in the Mfuzz R package (Kumar and Futschik, 2007), which is insensitive to noise, was performed to mine the transcriptomic modules of the DEGs.

## Results and Discussion

### Sample Correlation and PCA Analysis of the *M. Sextelata* Transcriptome

**FIGURE 1:**
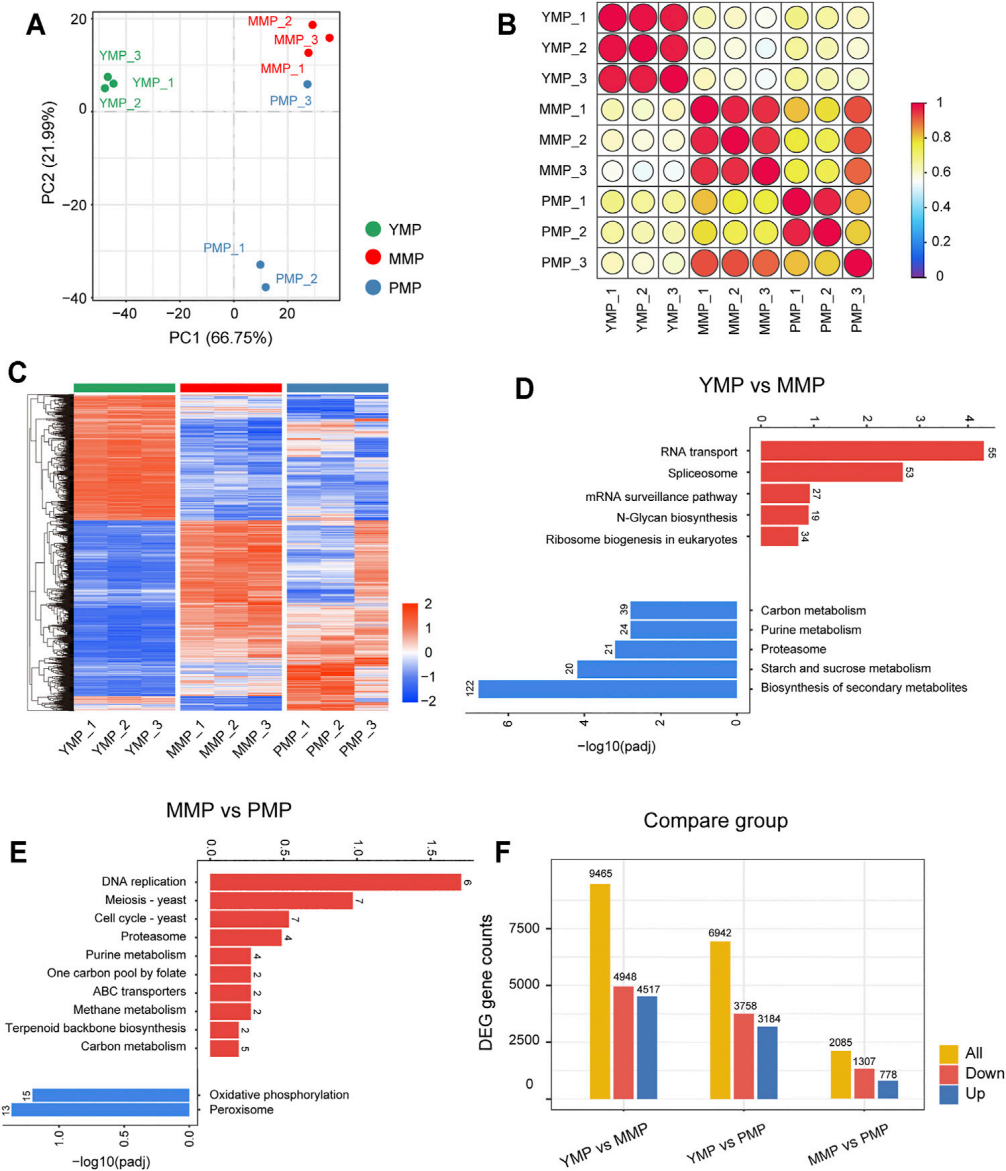
Dynamic transcriptional changes during three growth stages of *M. sextelata*. **(A)**: PCA analysis of the samples from the three stages. **(B)**: The correlation heat map of the samples from the three stages. **(C)**: The heat map showing the differentially expressed genes among the three stages (the red and blue colors indicate the pathways enriched by the upregulated and downregulated genes, respectively). Bar plots representing the KEGG enrichment results (the red and blue bars indicate the pathways enriched by the upregulated and downregulated genes, respectively) for **(D)**: YMP vs. MMP and **(E)**: MMP vs. PMP. **(F)**: The number of differentially expressed genes at the three different stages, i.e., the young mushroom period (YMP), marketable mature period (MMP), and physiological maturity period (PMP).

To investigate the expression dynamics during different sclerotial development stages, samples from the three developmental stages, i.e., the YMP, MMP and PMP, were collected for the RNA sequencing. PCA analysis of the gene expression profiles indicated that the first (PC1) and second (PC2) principal components accounted for 66.75 and 21.99% of the total variance, respectively. The results showed that the samples obtained from the three developmental stages were divided into three distinct regions (Figure 1A). This implied that the transcriptome showed marked changes during the different developmental stages of *M. sextelata*, and that the transcriptome may control phenotypic changes during development. The Spearman correlation coefficients of the samples were calculated based on the gene expression profiles, i.e., the abundance of gene expression, in each sample. The correlation was mapped to the respective colors in the form of a bubble heat map (Figure 1B). In addition, the correlation matrix revealed that there were significant differences between the transcriptomes at the different stages, and the similarity between the transcriptomes at the different stages was high. This not only confirmed the differences in the transcriptomes, but also showed the high sample quality and stability of the sequencing results. Genes with an adjusted *p*-value<0.05 and |log2FoldChange|≥1.0 were presented in the form of heat map (Figure 1C). The DEGs from nine samples were divided into specific clusters, as illustrated by the cluster dendrogram, hinting that these genes were involved in specific phenotypes at different developmental stages.

### Dynamic Transcriptional Changes During the Three Development Stages of *M. Sextelata*

The upregulated or downregulated differential DEGs of *M. sextelata* were enriched in the KEGG pathway analysis. The enrichment results for the YMP, compared with the MMP stage (YMP vs. MMP) and MMP compared with the PMP stage (MMP vs. PMP) were presented as bar graphs (Figures 1D–F). The functional annotation of DEGs indicated that the stage-specific genes were involved in the metabolic or development-related pathways; with the growth of *M. sextelata*, the pathways were activated or inhibited procedurally. The DEGs upregulated during the YMP were significantly enriched in the N-glycan biosynthesis pathway. Previous studies have reported that N-glycans are essential for the development and reproduction of rice (Veit et al., 2015), and were also related to the root growth of *Arabidopsis thaliana* (Liebminger et al., 2010). This implied that N-glycans play a key role in the development of *M. sextelata* during the juvenile period. Compared with the juvenile period and the PMP, carbon metabolism was highly activated in the immature commodity fruiting body period. Carbon catabolism provides fungi with energy in the form of reducing equivalents and ATP, as well as essential precursor metabolites for the biosynthesis of essential metabolites (Patyshakuliyeva et al., 2013). Previous studies on mushrooms have shown that the rate and flow of carbon metabolism during fruiting body development are high (Wells et al., 1987; Claydon et al., 1988; Patyshakuliyeva et al., 2013) and that the degradation of xylan is decreased at the end of the mushroom production cycle (Waksman and Nissen, 1932; Wood and Leatham, 1983). This is consistent with the activation of carbon metabolism during the MMP and inhibition of carbon metabolism during the PMP in morels, implying that *M. sextelata* also followed this carbon metabolism pattern. Meanwhile, purine metabolism, which showed the same trend from the YMP to the PMP, has been proved to provide tolerance to plants against various stresses, regulate their growth (Kawagishi, 2021), and remain at a high levels during the fruiting body growth of *Lentinula edodes* (Wang et al., 2018). The proteasome directly affects the renewal of certain proteins that are involved in cell cycle control and cell apoptosis and was found to be associated with fruiting body development (Yamada et al., 2006). In order to facilitate future research regarding this aspect, the genes involved in significant pathways associated with this process are listed in Supplementary Table S1. The numbers of DEGs that were upregulated or downregulated in different pairwise comparisons were represented using bar plots (Figure 1F). All these results showed the dynamic transcriptome panorama during the three stages of *M. sextelata* growth. Gene ontology analysis also have been conducted with transcriptomics data, but it didn’t detect any significantly term (Supplementary Figure S2 and Supplementary Table S1).

### PCA Analysis of Metabolic Samples From *M. Sextelata*

**FIGURE 2:**
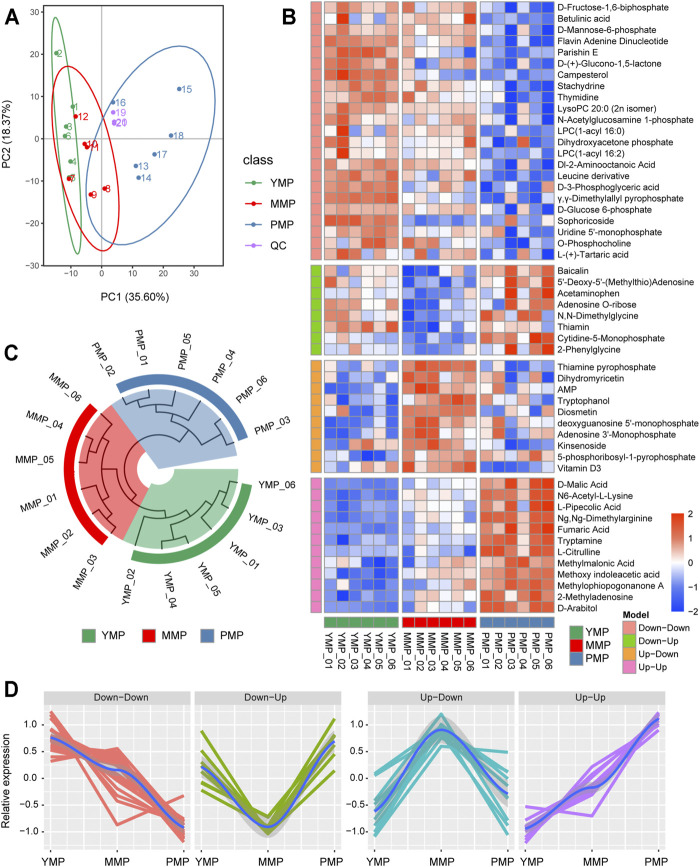
Dynamic metabolomics changes during the three growth stages of *M. sextelata*. **(A)**: PCA analysis of the samples during the three stages and the QC reference. **(B)** The heatmap of metabolites showing distinct patterns among the three growth stages. The time-dependent trend analysis divides the metabolites into four different patterns: Up−Up, Up−Down, Down−Up, and Down−Down. **(C)** The circular clustering tree consists of nine samples, considering differential metabolites as the feature vector. **(D)** A line chart showing the relative expression patterns of the potential marker metabolites of each cluster.

To investigate the dynamic process of metabolism during the different developmental stages of *M. sextelata*, samples from the three developmental stages, i.e., the YMP, MMP and PMP, were collected for metabolomics analysis. A total of 394 metabolites (150 and 244 detected in the negative-ion and positive-ion mode, respectively) were detected by HPLC-MS/MS. PCA analysis showed that the first (PC1) and second (PC2) principal components accounted for 35.60 and 18.37% of the total variance, respectively. The samples from the three developmental stages of *M. sextelata* were divided into three distinct regions based on the transcriptional characteristics (Figure 2A), consistent with findings from a previous metabolome-based research (Deng et al., 2021). This indicated that the changes of metabolites in the different developmental stages of *M. sextelata* were very notable, consistent with the results of the transcriptome analyses, suggesting that metabolome data can be used to study the developmental phenotypic changes of *M. sextelata*. Further, three QC samples were used to monitor the stability of the analysis. The high correlation among the QC samples indicates that the detection method is stable and can be used for subsequent analysis.

### Identification of Time-Dependent Metabolite Modules of *M. Sextelata*

There were 53 differentially expressed metabolites identified based on the empirical Bayesian mixing model; these were presented in the form of heat map (Figure 2B). These differential metabolites could be divided into four different patterns with dynamic changes and were associated with the growth of *M. sextelata*. In the Down−Down pattern, the levels of metabolites, including some carbohydrate metabolites, such as D-fructose-1,6-biphosphate, D-mannose-6-phosphate, and D-(+)-glucono-1,5-lactone, decreased during the growth of *M. sextelata*. This hinted that a large amount of carbohydrates was stored during the YMP, and their levels decreased with the growth and development of *M. sextelata*. Further, the mechanism underlying plant growth regulation depends on the acquisition of sugar *via* the signal transduction pathway (Van den Ende, 2014). In addition, uridine 5′-monophosphate and thymidine are involved in pyrimidine metabolism, which is related to arginine biosynthesis in morels (Zrenner et al., 2006). N,N-Dimethylglycine, which was involved in the Down−Up pattern, was upregulated during the YMP and downregulated during the MMP. This was consistent with the findings of a previous research, which reported that the energy requirements during the YMP were met by the oxidation of amino acids and the degradation of other storage compounds, until the photosynthetic machinery is fully functional (Galili et al., 2014). In addition, free amino acids were related to the flavor of mature morels (Wang et al., 2019). This provided physiological clues regarding the source of nutrition for *M. sextelata* during the YMP and the flavor during the PMP. Dihydromyricetin and adenosine monophosphate, which were involved in the Up−Down pattern, were upregulated during the MMP and were involved in the biosynthesis of secondary metabolites; this could mediate ecological interactions, which may give the organism a selective advantage by improving its viability or fecundity. These metabolic molecules may have a protective effect on *M. sextelata* during the MMP. In the Up–Up model, the levels of L-citrulline and fumaric acid, which serve as auxiliary molecules associated with arginine metabolism, gradually increased with the growth of *M. sextelata*. This pattern has also been found in previous studies (Deng et al., 2021), and arginine is an essential metabolite in many cells and developmental processes (Winter et al., 2015). Moreover, fumaric acid is an essential component of the tricarboxylic acid cycle (TCA), implying the energy enhancement during the PMP. Based on the differentially expressed metabolites observed using the three stages, the Euclidean distance was calculated, and then, the hierarchical clustering method was used to display the similarity between the samples in the form of circular tree (Figure 2C). The patterns of these significant changes can be used to cluster the samples well; accordingly, the clustering tree was divided into three main branches. The detailed information regarding the pattern of metabolite changes during the different growth stages of *M. sextelata* are listed in Supplementary Table S3. All these results revealed that the growth-associated metabolites were stage-specific.

### Transcriptional Basis of Dynamic Changes in Growth-Dependent Metabolites

**FIGURE 3:**
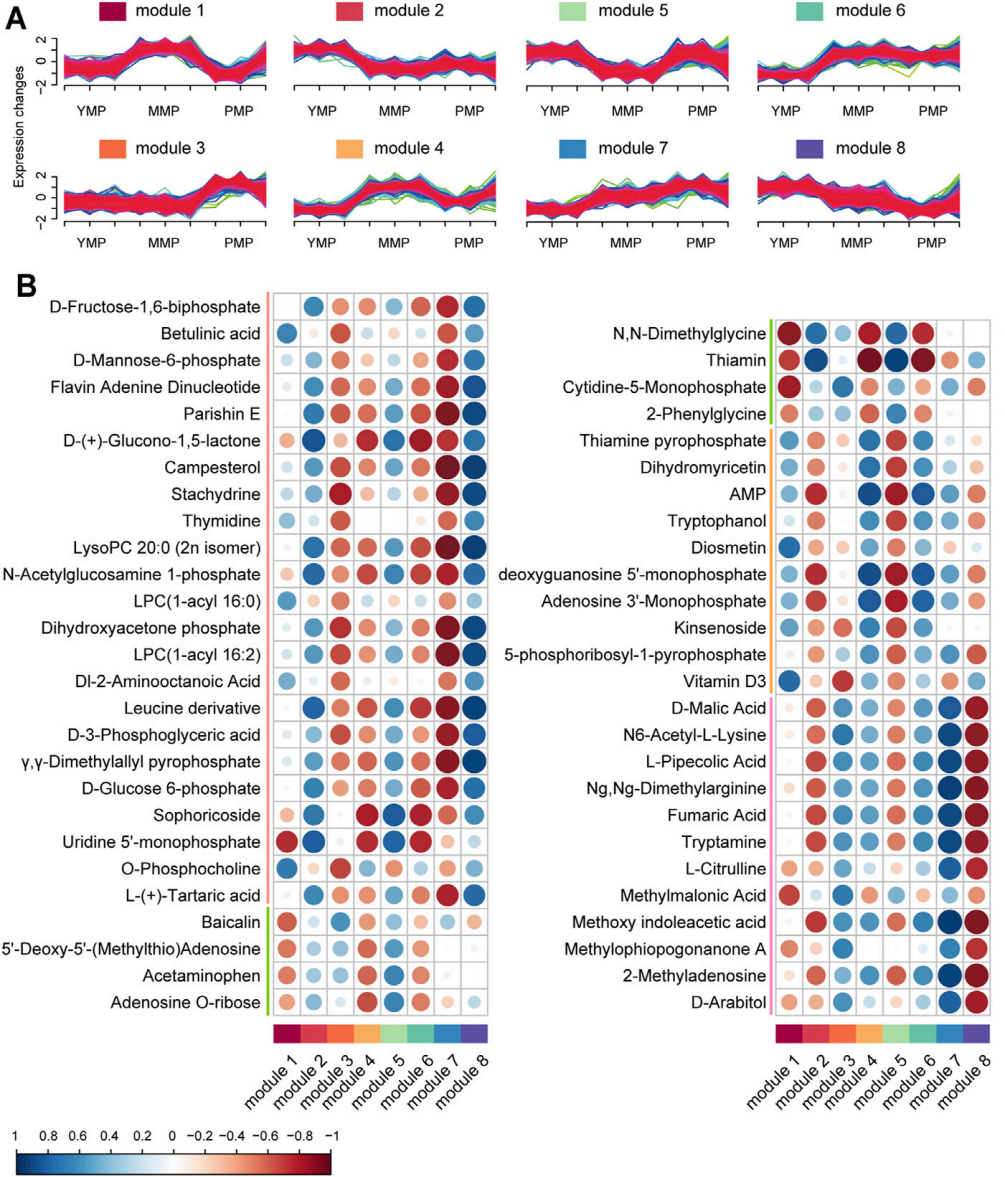
Joint analysis of dynamic changes in growth-dependent metabolites. **(A)** Modules of differentially expressed genes during the three growth stages of *M. sextelata*, as obtained using fuzzy c-means clustering. **(B)** Bubble map showing the module–metabolite correlations. The blue and red colors indicate positive and negative correlations, respectively. Each column corresponds to a module indicated by different colors.

Based on the unsupervised clustering method, a total of eight functional modules were identified; the genes in these modules showed different expression patterns (Figure 3A, Supplementary Table S4). The genes in module 1 tended to be highly expressed during the MMP, while those in modules 7 and 8 showed mutually exclusive expression. It implied that distinct functional modules played their own specific roles during the different growth periods of *M. sextelata* and served to better explain the developmental physiological changes in this mushroom. To gain further insights into the physiological changes occurring during the different growth stages, the functional modules and metabolites were jointly analyzed, establishing a module-trait relationship network. The correlation bubble graph (Figure 3B) showed that modules 7 and 8, which showed opposite transcriptional expression trends, had a correlated pattern in the metabolite network. Module 7 was specifically related to carbohydrate metabolites, such as D-fructose-1,6-biphosphate and D-(+)-glucono-1,5-lactone and this module also contained enzymes such as α-glucosidase, glycoside hydrolase, and pentokinase, which play important roles in the process of sugar and glycoconjugates in organisms (Minic and Jouanin, 2006; Minic, 2008). Furthermore, protein tyrosine phosphatase and glucanase were included in module 8. Glycanase is unevenly distributed in plants and aids microspore development and fruit maturation. Protein tyrosine phosphatase performs important physiological functions in plant growth, organ development, signal transmission, and stress response. It was seen that module 8 was closely related to sugars and phosphoric acid. These results revealed that some specific enzymes played important roles in the growth process of *M. sextelata via* the regulation of the levels of metabolic molecules. Additionally, module 1 showed high positive relation with green trend which down-expressed in MMP and up-expressed in YMP and PMP periods which contained metabolic molecules, such as Baicalin, 5′-Deoxy-5'-(Methylthio)Adenosine and acetaminophen. Meanwhile, module 2 was highly similar with module 3 in the red metabolic molecule pattern but contrary in other metabolic pattern. In addition, module 4 and module 5 had a consistent contrary pattern across all metabolic molecule pattern. Furthermore, module 4 and module 6 which had distinct expression trend had a common impact on metabolic molecules.

## Conclusion

In this study, we explored the transcriptome and metabolome dynamics of *M. sextelata* during its growth process and identified the key genes, metabolites, and pathways involved in the vegetative growth of this fungus. At first, the results of the transcriptome analysis indicated that the metabolic activity of *M. sextelata* showed tremendous changes during the three growth stages, and these changes may be related to the phenotype. The metabolomics analysis also proved the existence of stage-dependent small molecules, such as D-fructose-1,6-biphosphate, D-mannose-6-phosphate, and D-(+)-glucono-1,5-lactone. Furthermore, joint analysis of the transcriptome and metabolome data revealed that the transcriptome may affect the metabolic molecules at different stages through some specific enzymes, such as α-glucosidase, glycoside hydrolase, and tyrosine phosphatase, serving important physiological functions associated with plant growth, organ development, and signal transmission. In summary, these results provide comprehensive insights into the dynamic processes associated with the vegetative growth of *M. sextelata* and new clues for optimizing the conditions for its cultivation.

## Data Availability

The datasets presented in this study can be found in online repositories. The names of the repository/repositories and accession number(s) can be found below: https://www.ncbi.nlm.nih.gov/bioproject/?term=PRJNA790711.
