# Supplementary figures and images for: Integration of Transcriptomics and Metabolomics for Understanding the Different Vegetative Growth in Morchella Sextelata
Source: Front Genet. 2022 Feb 4;12:829379. doi: 10.3389/fgene.2021.829379 (PMC8854800; doi:10.3389/fgene.2021.829379)

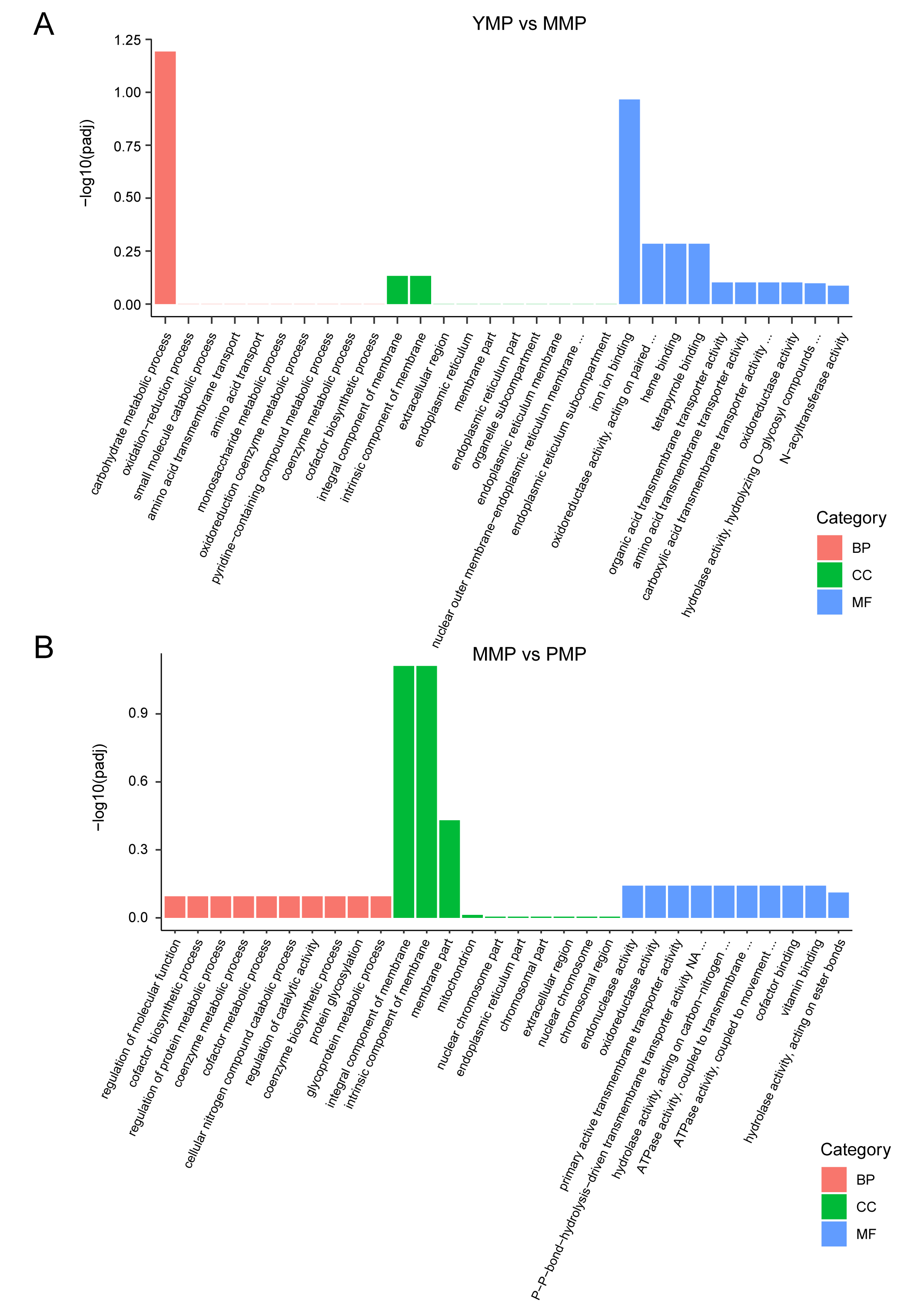

Supplement: Supplementary file 5 [file Image1.TIF]
